# 15-Day Duration of Venetoclax Combined with Azacitidine in Treatment-Naive Higher-Risk Myelodysplastic Syndromes: A Prospective Multicenter Study

**DOI:** 10.3390/cancers18010159

**Published:** 2026-01-02

**Authors:** Binbin Lai, Chen Mei, Xiao Yan, Lieguang Chen, Yi Wang, Lixia Sheng, Shanhao Tang, Liping Mao, Ping Zhang, Yongcheng Sun, Wanzhuo Xie, De Zhou, Wenyuan Mai, Huafeng Wang, Liya Ma, Yinjun Lou, Wenjun Wu, Huifang Jiang, Jin Zhang, Baodong Ye, Hongyan Tong, Guifang Ouyang

**Affiliations:** 1 Department of Hematology, The First Affiliated Hospital of Ningbo University, 59 Liuting Street, Ningbo 315000, China; binbinlai0213@163.com (B.L.); zhzxyx@163.com (X.Y.); fyychenlieguang@nbu.edu.cn (L.C.);; 2 Department of Hematology, The First Affiliated Hospital, Zhejiang University School of Medicine, 79 Qingchun Road, Hangzhou 310003, China; meichenblood@yeah.net (C.M.); ruicostas610@sina.com (D.Z.);; 3 Myelodysplastic Syndromes Diagnosis and Therapy Center, The First Affiliated Hospital, Zhejiang University School of Medicine, Hangzhou 310000, China; 4 Bone Marrow Transplantation Center, The First Affiliated Hospital, Zhejiang University School of Medicine, Hangzhou 310000, China; 5 Department of Hematology, Zhejiang Provincial Tongde Hospital, 234 Gucui Road, Hangzhou 310012, China; 6 Department of Hematology, Sir Run Run Shaw Hospital, Zhejiang University School of Medicine, 3 Qingchun East Road, Hangzhou 310016, China; 7 Department of Hematology, Zhejiang Provincial Hospital of Traditional Chinese Medicine, 54 Youdian Road, Hangzhou 310009, China

**Keywords:** higher-risk myelodysplastic syndromes, venetoclax, azacitidine, hematopoietic stem cell transplantation

## Abstract

Background: Higher-risk myelodysplastic syndromes (HR-MDS) carry a high risk of progression to acute myeloid leukemia and poor overall survival. Hypomethylating agents (HMAs), such as azacitidine, remain the standard of care but have limited efficacy. A 15-day venetoclax-azacitidine regimen has shown promising objective response rates (ORR) and potential as a bridge to allogeneic hematopoietic stem cell transplantation (HSCT) in relapsed/refractory HR-MDS. We conducted a prospective multicenter trial to evaluate its efficacy and safety in previously untreated patients. Methods: This multicenter prospective study enrolled treatment-naïve HR-MDS patients (IPSS-R > 3.5). Venetoclax was administered on days 1–15 (escalated from 100 to 400 mg), combined with azacitidine (75 mg/m^2^) on days 1–7 of each 28-day cycle. The primary endpoint was ORR (2006 IWG criteria); secondary endpoints included complete remission (CR), overall survival (OS), and AML progression. Results: Twenty-eight patients (median age: 63 years) were enrolled, with a median follow-up of 8.5 months. ORR was 85.7% per 2006 IWG (CR: 35.7%, marrow CR: 50.0%), and 78.6% per 2023 IWG (CR: 35.7%). Responses were consistent across molecular and IPSS-R subgroups. Median OS was not reached. High neutrophil count and high cytogenetic risk were favorable factors; TP53 mutation/deletion was an adverse prognostic marker. Grade 3–4 hematologic toxicities included neutropenia (96.4%), anemia (71.4%), and thrombocytopenia (64.3%). Serious adverse events (35.7%) were mainly infections. No dose-limiting or unexpected toxicities were observed. Conclusions: The 15-day venetoclax plus azacitidine regimen demonstrated high efficacy and manageable toxicity in treatment-naïve HR-MDS. It may be particularly beneficial for patients with high neutrophil counts, adverse cytogenetics, or those eligible for HSCT, supporting further investigation in larger trials.

## Simple Summary

This study evaluated the efficacy and safety of a 15-day venetoclax combined with azacitidine regimen in 28 untreated high-risk myelodysplastic syndromes patients. Results demonstrated that the regimen was highly effective, with an overall response rate of 85.7% and a complete remission rate of 35.7%. Consistent responses were observed across molecular and risk subgroups. The most common severe hematologic toxicity was neutropenia. The study confirmed that this combination regimen yields high response rates with manageable safety, and it may be particularly beneficial for patients with high neutrophil counts, adverse cytogenetics, or those eligible for hematopoietic stem cell transplantation, warranting further validation in larger trials.

## 1. Background

Myelodysplastic syndromes (MDS) represent a heterogeneous group of clonal myeloid disorders originating from hematopoietic stem cells. These disorders are characterized by ineffective hematopoiesis, bone marrow dysplasia and an increased risk of progression to acute myeloid leukemia (AML), with a median age of 76 years at diagnosis [1]. Higher-risk MDS, defined by an International Prognostic Scoring System (IPSS) score of ≥1.5 or a Revised IPSS (IPSS-R) score of >3.5, accounts for approximately 43% of all MDS cases [2]. Notably, the majority of MDS cases, particularly higher-risk patients, harbor multiple somatic mutations. These mutations not only significantly increase the risk of AML transformation but also contribute to higher overall mortality. The prognosis for patients with higher-risk myelodysplastic syndromes (HR-MDS) remains poor, with a median survival of only 1.6 years under current standard treatment [3].

The primary therapeutic goal for higher-risk MDS is to prolong patient survival, with the ultimate aspiration being cure [4]. Hematopoietic Stem Cell Transplantation (HSCT) remains the only potentially curative treatment for MDS [5,6]. However, due to limitations such as advanced age, comorbidities, and donor availability, HSCT is accessible to only a small subset of patients [7]. For the majority of HR-MDS patients, hypomethylating agents (HMAs) are the approved first-line therapy. These agents exert their effects primarily through inhibition of DNA methyltransferases, leading to transcriptional reactivation of silenced genes. Their cytotoxicity profile also plays a role in therapeutic outcomes [8,9,10]. While direct comparative studies between 5-azacitidine (AZA) and decitabine are limited, randomized clinical trials have shown that only 5-azacitidine improves patient survival [11,12]. Notably, the AZA-001 phase III trial demonstrated that azacitidine significantly extended median Overall Survival (mOS) to 24.5 months in HR-MDS patients, compared to 15 months with conventional treatment [12]. This has established 5-azacitidine as the standard of care for high-risk MDS. However, real-world evidence and subsequent analyses have revealed a more modest mOS range of 14–19 months with azacitidine [13], highlighting the critical need for novel therapeutic strategies to improve outcomes in HR-MDS patients.

Combination therapies with azacitidine have emerged as a key research focus, aiming to enhance efficacy, prolong remission, delay disease progression, and create bridging opportunities for HSCT in more patients. Venetoclax, an oral small-molecule inhibitor of the anti-apoptotic protein BCL-2, has shown promise in inhibiting the release of pro-apoptotic proteins and inducing apoptosis in malignant cells. It is approved in combination with azacitidine for newly diagnosed AML patients who are unfit for intensive chemotherapy [14,15,16]. Given the biological similarities between higher-risk MDS and AML, venetoclax has been tested for its ability to induce apoptosis in higher-risk MDS [17]. Our preliminary real-world data demonstrated that the 15-day venetoclax-azacitidine regimen achieved an encouraging objective response rate and showed potential for reducing disease burden prior to allogeneic HSCT in relapsed/refractory HR-MDS patients [18]. Furthermore, results from an open-label, multi-center, phase 1b study (NCT02942290) showed that the treatment was well tolerated, along with favorable efficacy outcomes [19]. Based on these findings, we initiated an open-label, prospective multicenter clinical trial (ChiCTR2200056993) to evaluate the efficacy and safety of a 15-day venetoclax plus azacitidine (VA) regimen in patients with treatment-naive HR-MDS. Here we report the key results of this study.

## 2. Methods

### 2.1. Patients

This study enrolled previously untreated HR-MDS patients (aged ≥18 years) from five participating institutions from China. Eligible patients met the following inclusion criteria: bone marrow aspiration/biopsy confirming a bone marrow blast percentage of <20% during screening, an IPSS-R score > 3.5, an Eastern Cooperative Oncology Group (ECOG) performance status ≤ 3, and adequate hepatic and renal function to tolerate the venetoclax-Azacitidine (VA) regimen. Patients scheduled for immediate allogeneic HSCT were excluded. Additional exclusion criteria included a diagnosis of myelodysplastic/myeloproliferative neoplasms, active malignancies, a life expectancy of <6 months, and uncontrolled infections. The study was conducted in accordance with the ethical principles of the Declaration of Helsinki and was approved by the institutional ethics committees of all participating centers. Written informed consent was obtained from all patients or their legal representatives before study enrollment.

### 2.2. Procedures

Patients received venetoclax, initiated at 100 mg daily and escalated to 400 mg over three days, from days 1 to 15 of each 28-day cycle, in combination with standard-dose azacitidine (75 mg/m^2^ on days 1–7 of each cycle). Antibiotic prophylaxis was mandated for all patients during the initial cycles and continued for those experiencing grade 3 neutropenia. Investigators were permitted to administer granulocyte colony-stimulating factor (G-CSF) to manage febrile neutropenia or neutropenic sepsis. The venetoclax dosage was adjusted for patients receiving concomitant CYP3A inhibitors, particularly azole antifungals. For patients on moderate CYP3A inhibitors (e.g., isavuconazole), the venetoclax dose was reduced by 50%, whereas for those on strong CYP3A inhibitors (e.g., voriconazole, posaconazole), the dose was reduced by 75%, as per established guidelines. Treatment was not interrupted for clinically insignificant cytopenia, such as neutropenia or thrombocytopenia, but could be suspended in cases of grade 4 febrile neutropenia or grade 4 thrombocytopenia with life-threatening bleeding. Efficacy assessments were performed after Cycle 2, and treatment cycles were repeated every 4–6 weeks until disease progression, intolerable toxicity, patient withdrawal, or HSCT. Final follow-up was completed in January 2024 through clinic visits or telephone interviews. At baseline, bone marrow single-cell suspensions were analyzed for mutations in 27 of 28 patients (96.4%). Genomic DNA was extracted from bone marrow cells, amplified using AmpliSeq multiplex PCR (Thermo Fisher Scientific, Waltham, MA, USA), and sequenced using next-generation sequencing (NGS) on the NovaSeq 6000 platform (Illumina, San Diego, CA, USA). Sequencing data were interpreted using a dedicated hematology-oncology database. FLT3 internal tandem duplication (ITD) mutations were identified by capillary electrophoresis fragment analysis and NGS, performed at Wuhan Kangshengda Medical Testing Laboratory. The average sequencing depth was 2000×, with a variant allele frequency (VAF) > 1% considered positive. Following two cycles of induction therapy, 11 patients proceeded to allogeneic HSCT.

### 2.3. Safety Assessment

Adverse events (AEs) were monitored through outpatient visits, laboratory tests, and during hospitalization. AEs were graded and documented according to the National Cancer Institute Common Terminology Criteria for Adverse Events (NCI CTCAE v5.0).

### 2.4. Endpoints and Evaluation

The efficacy of MDS treatment was evaluated through bone marrow analysis at the end of each treatment cycle. The primary efficacy endpoint was overall response rate (ORR), assessed according to the modified International Working Group (IWG 2006) criteria [20]. ORR was defined as the proportion of patients achieving complete remission (CR), partial remission (PR), or marrow complete remission (mCR). In addition, the revised 2023 International Working Group (IWG) criteria for high-risk MDS were incorporated for supplementary analysis Under the IWG 2023 criteria [21], ORR encompasses CR rate, PR rate, and CR with limited hematologic recovery (CRL), which includes unilineage CR (CRuni) and bilineage CR (CRbi). Furthermore, ORR also includes CR with partial hematologic recovery (CRh) and hematologic improvement (HI), which is further categorized into erythroid (HI-E), platelet (HI-P), and neutrophil (HI-N) improvements. Key secondary endpoints included CR rate, OS, and disease progression rate. OS was defined as the time from treatment initiation to death from any cause, with data from patients alive at the cutoff date censored at the last recorded follow-up.

### 2.5. Statistical Analysis

Continuous variables were summarized as median values with interquartile ranges (IQR). Categorical variables were described as counts and percentages. The chi-square test or Fisher’s exact test was used to compare response rates between subgroups. Logistic regression analyses were performed to explore baseline factors associated with achieving an overall response or a CR. OS was analyzed by the Kaplan–Meier method and log-rank tests for subgroup comparisons. Cox proportional hazards modeling was used to identify independent predictors of OS. Univariate variables with a *p*-value < 0.2 were included in the multivariate analysis. A two-tailed *p* < 0.05 was considered statistically significant. Statistical analyses were carried out using IBM SPSS Statistics (v27), and figures were generated with GraphPad Prism (v9.0).

## 3. Results

### 3.1. Patient Demographics and Disease Characteristics

A total of 28 untreated HR-MDS patients were enrolled in the study. Baseline characteristics, including age, gender, ECOG score, bone marrow blast percentage, IPSS-R risk category, cytogenetic profile, transfusion dependence, and mutation status, were comprehensively documented. The median age of the cohort was 63 years (IQR: 59–68 years), with a predominance of male patients (18/28, 64.3%). At the initiation of combination therapy, the median bone marrow blast percentage was 12.0% (IQR: 9.75–13.25%). Based on the IPSS-R scoring system, 57.1% (16/28) of patients were categorized as very high risk, 25.0% (7/28) as high risk, and 17.9% (5/28) as intermediate risk. Notably, 82.1% (23/28) of the cohort fell into the high-risk category. Meanwhile, based on the IPSS-M scoring system, 64.3% (18/28) of patients were classified as very high risk, 28.6% (8/28) as high risk, and 7.1% (2/28) as moderate-high risk. Molecular profiling (27 of 28 patients) showed that the frequently mutated genes were ASXL1 (8/27,29.6%), DNMT3A (8/27, 29.6%), and TP53 mutation/deletion (7/27, 25.9%). Other recurrent mutations included TET2, RUNX1, NPM1 and IDH1/2 (each in 4 patients, 14.8%). Only one patient (3.6%) had no detectable mutation. These findings reflect the adverse genetic background of the cohort. Detailed mutation data are presented in Table 1.

### 3.2. Treatment Response

**Figure 1:**
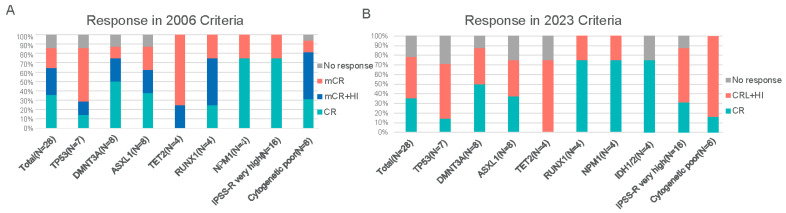
Treatment responses to the 15-day VA regimen based on IWG criteria. (**A**) Best responses according to the 2006 International Working Group (IWG) criteria for MDS. Categories include complete remission (CR), marrow complete remission (mCR), partial remission (PR), and hematologic improvement (HI). (**B**) Best responses according to the 2023 IWG criteria for high-risk MDS. Categories include CR, PR, and CR with limited hematologic recovery (CRL), as well as responses with hematologic improvement (HI). Abbreviations: CR, complete remission; mCR, marrow complete remission; PR, partial remission; CRL, complete remission with limited recovery; HI, hematologic improvement.

After receiving two cycles of 15-day venetoclax combined with Azacitidine, the overall response rate (ORR) was 85.7% according to the IWG 2006 criteria. Specifically, 35.7% (*n* = 10) of patients achieved CR, while 50.0% (*n* = 14) achieved marrow mCR, with no cases of PR (Figure 1A). Among the 14 patients with mCR (per IWG 2006 criteria), 57.1% (8/14) also demonstrated hematologic improvement (HI). The total proportion of patients achieving CR or mCR with HI was 64.3% (*n* = 18). According to the IWG 2023 criteria, the ORR was 78.6%, with 10 patients achieving CR and 12 patients attaining CR with limited hematologic recovery (CRL) or hematologic improvement (HI) (Figure 1B). The median time to CR was 2.3 months. Reasons for treatment discontinuation: HSCT (39.3%), progressive disease (17.9%), adverse event (14.3%), and patient withdrawal (10.7%). Of the 28 evaluable patients, 5 cases (17.9%) progressed to acute myeloid leukemia (AML), with a median time to progression of 3.2 months.

### 3.3. Subgroup Analysis

The VA regimen demonstrated consistently high overall remission rates across all analyzed subgroups, including those defined by IPSS-R risk categories, cytogenetic profiles, or specific mutations. Notably, among patients with adverse TP53 mutations or deletions, ORR was 85.7% (6/7). ORR was similarly high in those with DNMT3A or ASXL1 mutations (87.5% each, 7/8 patients). All evaluable patients (4/4, 100%) with mutations in TET2, RUNX1, NPM1, or IDH1/2 responded to therapy. Complete remission rates varied by mutation subtype: for example, only 14.3% of TP53-mutated patients achieved CR, versus 50.0% of DNMT3A-mutated and 37.5% of ASXL1-mutated patients, and 75.0% of NPM1- or IDH1/2-mutated patients achieved CR. Despite these numerical differences, the study was not powered to detect statistically significant response differences between subgroups. Neither univariate nor multivariate logistic regression identified any baseline demographic, clinical, or genetic factor as a significant predictor of achieving an overall response or CR (Table 2). This lack of detected differences likely reflects the limited sample size.

### 3.4. Overall Survival

**Figure 2:**
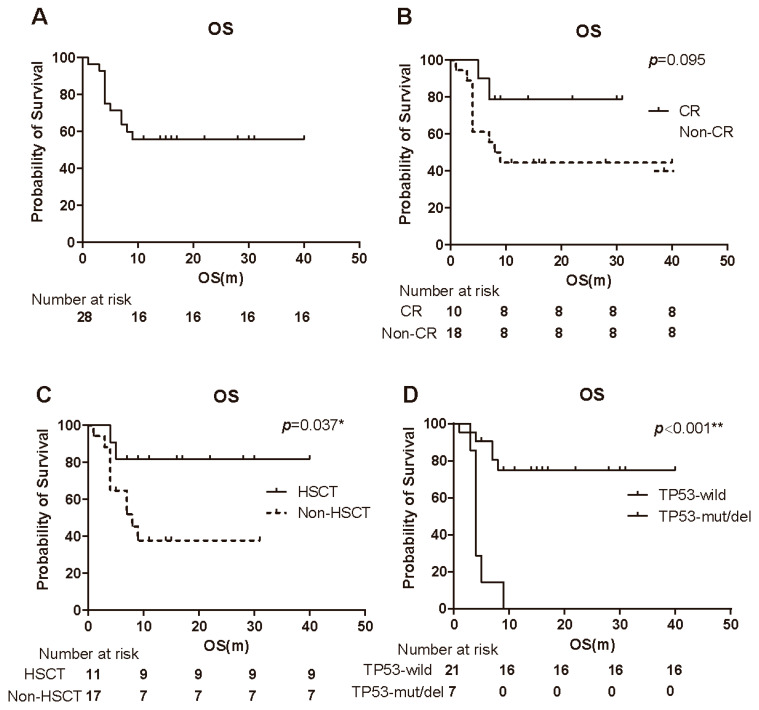
Kaplan–Meier survival analyses of patients treated with 15-day venetoclax plus azacitidine. (**A**) Overall survival (OS) of all 28 patients (median follow-up 8.5 months). The median OS was not reached. (**B**) OS stratified by response status: patients achieving CR versus those not achieving CR. There was a non-significant trend toward longer OS in the CR group (*p* = 0.095). (**C**) OS comparison between patients who underwent HSCT (after responding to therapy) and those who did not undergo transplantation. Post-transplant patients had significantly improved OS (*p* = 0.037 by log-rank). (**D**) OS comparison between patients with TP53 mutations/deletions and those with wild-type TP53. TP53-aberrant patients had significantly shorter OS (*p* < 0.001). Abbreviations: OS, overall survival; CR, complete remission; HSCT, hematopoietic stem cell transplantation. * *p* < 0.05; ** *p* < 0.01.

With a median follow-up of 8.5 months, 42.9% (12/28) of patients had died, while 57.1% (16/28) remained alive. The median OS for the entire cohort was not reached at the time of the analysis (Figure 2A). There was no significant difference in OS between responders and non-responders (Figure 2B). Although the difference in OS between the CR group and the non-CR group did not reach statistical significance (log-rank test, *p* = 0.095), the CR group showed a trend toward prolonged survival (not reached vs. 8.5 months; Figure 2B). Notably, patients who underwent HSCT exhibited superior survival compared to those who did not receive transplantation (log-rank test, *p* = 0.037; Figure 2C). Additionally, patients with TP53 mutations or deletions had significantly shorter OS compared to those without these genetic abnormalities (log-rank test, *p* < 0.001; Figure 2D).

### 3.5. Prognostic Factors

We performed multivariate Cox regression to identify independent prognostic factors for OS (Table 3). In univariate Cox regression analysis, BM blast percentage treated as a categorical variable was potentially associated with improved survival (*p* = 0.018). In this model, the presence of a TP53 mutations or deletions remained the strongest adverse factor, with a hazard ratio (HR) of 128.9 (95% CI 4.832–3438.954) and *p* = 0.004. Conversely, high-risk cytogenetics by IPSS-R was associated with improved survival (HR 0.069, 95% CI 0.005–0.871; *p* = 0.037) in our cohort. A higher baseline neutrophil count was also associated with prolonged OS (HR 1.413 per unit increase, 95% CI 1.066–1.872; *p* = 0.016). These two factors emerged as unexpected protective correlates of OS in the multivariate analysis. In contrast, undergoing HSCT showed a protective effect in univariate analysis (log-rank *p* = 0.037) but did not remain significant in the Cox model (*p* = 0.059), likely due to the influence of other covariates and the small sample size.

### 3.6. Safety

All 28 patients experienced at least one treatment-related AE (Table 4). As expected, hematologic toxicities were ubiquitous. Grade 3–4 neutropenia occurred in 96.4% of patients (*n* = 27) and grade 3–4 leukopenia in 92.9% of patients (*n* = 26), often reflecting the on-target myelosuppressive effect of the regimen. Grade 3–4 anemia and thrombocytopenia occurred in 71.4% (*n* = 20) and 64.3% (*n* = 18) of patients, respectively. Febrile neutropenia was documented in 50.0% patients (*n* = 14). The predominant serious AEs were infections: 35.7% of patients (*n* = 10) developed pneumonia (grade ≥ 3), and one patient (3.6%) developed sepsis. Non-hematologic AEs were generally low-grade (grade 1–2). The most common were gastrointestinal disturbances, for example, nausea in 46.4% (*n* = 13) and constipation in 32.1% of patients (*n* = 9). Other non-hematologic AEs included transient hepatic injury (10.7%, *n* = 3), renal function changes (10.7%, *n* = 3), and cardiac events (10.7%, *n* = 3), all predominantly grade 1–2. The 30-day and 60-day mortality rates after treatment initiation were 0% (*n* = 0) and 3.6% (*n* = 1), respectively, and no deaths were attributed to treatment-related toxicity. Notably, no unexpected treatment-emergent adverse events were observed, and no tumor lysis syndrome (TLS) occurred during the venetoclax ramp-up phase.

## 4. Discussion

This prospective multicenter clinical study was the first to explore the efficacy and safety of 15-day duration venetoclax in combination with azacitidine for the previously untreated HR-MDS patients.

The patients in our cohort had a median age of 63 years and were predominantly very high or high risk by IPSS-R, reflecting a population with poor prognosis. The treatment regimen demonstrated a remarkable ORR of 85.7%, showing robust efficacy even in patients with high or very high IPSS-R risk and adverse cytogenetic features, which consistent with the phase Ib trail [19]. Notably, with a median follow-up of 8.5 months, the median OS has not yet been reached. While longer follow-up is needed, this initial survival outcome appears favorable when compared to historical outcomes with azacitidine alone.

Due to the limited sample size, neither univariate nor multivariate analyses identified age, gender, blood counts, IPSS-R risk classification, or bone marrow blast percentage as significant predictors of remission rates. OS remains the gold standard for evaluating treatment efficacy in HR-MDS. Achieving CR was not statistically associated with better OS in our cohort, although there was a trend toward longer survival in patients who attained CR (*p* = 0.095 by log-rank test). This trend aligns with observations in other studies suggesting CR can be associated with improved survival in HR-MDS [22]. In our study, however, 11 of 28 patients proceeded to HSCT, including some who had not achieved CR. Our data suggest that successful HSCT bridging in non-CR patients may have attenuated the observed survival differential between CR and non-CR cohorts, potentially explaining the lack of significant OS benefit associated with CR attainment in survival analysis.

In our study, TP53 abnormalities, including mutations and deletions, were identified as the strongest adverse prognostic factor for OS, although they did not significantly affect OR and CR rates. At the data cutoff, the vast majority of patients with TP53 abnormalities had succumbed to disease progression or infectious complications. except for one who was lost to follow-up. Patients with HR-MDS harboring TP53 abnormalities generally exhibit resistance to standard therapies and poor prognosis. Moreover, the landmark VIALE-A trial investigating VA in newly diagnosed AML revealed striking differential responses based on TP53 status: whereas no responses (0%) were observed in TP53-mutated patients receiving azacitidine monotherapy, the VA regimen achieved a response rate of 55.3% (95% CI, 38.3–71.4) in this high-risk population [16]. Although no significant improvement in overall survival was observed, the response rate in TP53 mutated myeloid neoplasm patients reported in the study is consistent with our findings. These findings emphasize the urgent need for novel combination therapies targeting HR-MDS patients with TP53 mutations to improve survival outcomes. Our study suggests that the VA regimen may serve as an effective bridging therapy for HSCT in TP53-mutated patients, but optimal strategies to mitigate post-transplant complications in this high-risk group are still needed.

An unexpected finding in our analysis was that high-risk cytogenetics (as defined by IPSS-R) was associated with better OS in the multivariate model. Traditionally, high cytogenetic risk has been associated with poor prognosis according to IPSS-R or IPSS. In our study, this observation may be explained by the strong clinicopathological association between TP53 mutations and high-risk cytogenetic abnormalities, where the overwhelmingly poor prognosis driven by TP53 mutations likely confounds the independent prognostic value of cytogenetic risk stratification. In other words, among patients without TP53 abnormalities, those with adverse cytogenetics still fared reasonably well on the VA regimen. Consistent with this finding, the clinical study has demonstrated that the combination of venetoclax and decitabine achieves a significantly higher composite CRc in young AML patients with molecular adverse-risk disease compared to idarubicin and cytarabine, with rates of 91% and 42%, respectively [23]. Therefore, although high cytogenetic risk has historically conferred poor prognosis in the pre-targeted therapy era, the VA regimen may potentially improve clinical outcomes in high cytogenetic risk patients without TP53 mutations, mitigating what would historically be a very poor prognosis. This hypothesis will need validation in larger studies.

Our multivariable analysis also suggested that patients with higher baseline neutrophil counts had longer survival. We speculate that a preserved neutrophil count at diagnosis may indicate better underlying bone marrow reserve and immune function, enabling patients to tolerate the myelosuppressive VA therapy with fewer life-threatening infections. Venetoclax plus azacitidine is known to cause profound myelosuppression [12], so patients with a robust baseline neutrophil count could have more cushion to endure treatment. This association must be interpreted cautiously given the small sample, but it raises an interesting point about patient selection: HR-MDS patients with severe baseline neutropenia might be at higher risk for complications on this intensive combination. Extended follow-up and larger cohorts will be needed to confirm the protective impact of baseline neutrophils.

The combination of venetoclax and azacitidine has been shown to have significant myelosuppressive effects (12), likely due to the synergistic action of both agents. While AML patients typically receive venetoclax for 28 days [16], a shorter 15-day regimen was used in this study. This was based on the understanding that HR-MDS patients are often older, have baseline cytopenias due to ineffective hematopoiesis at diagnosis, and frequently present with multiple comorbidities. The decision to limit venetoclax to 15 days each cycle was intended to balance efficacy with safety in this vulnerable population. This approach appears to have been effective: while nearly all patients experienced cytopenias, these were manageable with supportive care, and early mortality was very low (no deaths within 30 days of treatment, and one death by 60 days from infection unrelated to therapy).

Allogeneic hematopoietic stem cell transplantation remains the curative treatment and standard of care for eligible patients. However, many higher-risk MDS patients cannot tolerate HSCT due to advanced age and comorbidities [3]. Recent advancements in reducing toxicity and managing infections have improved post-transplantation outcomes, making HSCT a recommended option for patients with suitable donors [24]. Prior studies have shown that, among patients undergoing allogeneic HSCT, those treated with HMA plus venetoclax had a two-year OS rate of 91%, compared to 51% in the HMA monotherapy group [25,26]. In our study, 39.3% (11/28) of patients underwent HSCT following VA therapy, with most surviving. Survival analysis also showed that, compared to non-HSCT patients, those who underwent HSCT after VA therapy had significantly improved OS. These findings underscore the potential of VA regimens in rapidly inducing remission, allowing for early evaluation for HSCT and facilitating bridging to transplantation.

However, we acknowledge the limitations of our study. The absence of a control group in this trial limits the reliability of efficacy conclusions. Additionally, the limited sample size necessitates validation in larger cohorts to confirm these observations regarding VA regimen efficacy across different subgroups. Although the phase 3 VERONA trial [27], which enrolled some intermediate-risk patients, did not demonstrate an overall survival (OS) benefit with the VA regimen, it achieved superior response rates compared to the control. Moreover, certain subgroups, such as patients aged < 75 years, those with high blast counts, and those with high IPSS-R scores, showed a trend toward benefit. These findings regarding response rates are consistent with the conclusions of our study. Furthermore, our research indicated an OS benefit for EB2 patients in univariate analysis and for high-risk patients in multivariate analysis, observations that align with the VERONA study. Therefore, it is unlikely that the VA regimen will benefit all high-risk MDS patients. Future efforts should focus on identifying clinical characteristics (e.g., younger age, high blast count, high-risk stratification) and biomarkers (e.g., NPM1, IDH1/2, DDX41 mutations) to guide the application of the VA regimen in specific patient subgroups.

## 5. Conclusions

In summary, this prospective multicenter clinical study provides preliminary evidence supporting the efficacy and safety of a 15-day venetoclax regimen in combination with azacitidine for untreated HR-MDS. The combination produced a high ORR with manageable toxicity, even among patients with traditionally poor risk features. Our study shows the regimen may benefit patients exhibiting high baseline neutrophil counts, high cytogenetic risk, or those being considered for HSCT.

## Figures and Tables

**Table 1:** Patient demographics and disease characteristics.

Characteristics	N (%)
**Age, median (IQR), years**	63 (59–68)
**<65, n (%)**	16 (57.1%)
**≥** **65, n (%)**	12 (42.9%)
**Male, n (%)**	18 (64.3%)
**ECOG PS, n (%)**	
**0**	1 (3.6%)
**1**	18 (64.3%)
**2**	9 (32.1%)
**IPSS-R prognostic score, n (%)**	
**Intermediate**	5 (17.9%)
**High**	7 (25.0%)
**Very high**	16 (57.1%)
**Bone marrow blast category, n (%)**	
**5–9.5%**	6 (21.4%)
**10–19.5%**	22 (78.6%)
**Bone marrow blast count, median IQR)**	12 (9.75–13.25)
**Baseline transfusion dependence** **, n (%)**	
**RBC**	6 (21.4%)
**Platelet**	2 (7.1%)
**Cytogeneticrisk** **, n (%)**	
**Very good**	1 (3.6%)
**Good**	14 (50.0%)
**Intermediate**	7 (25.0%)
**Poor**	1 (3.6%)
**Very poor**	5 (17.9%)
**IPSS-M risk, n (%)**	
**Very high**	18 (64.3%)
**High**	8 (28.6%)
**Moderate high**	2 (7.1%)
**Baseline mutations, n (%)**	
**No mutations detected**	1 (3.6%)
**DMNT3A**	8 (29.6%)
**ASXL1**	8 (29.6%)
**TP53**	7 (25.9%)
**TET2**	4 (14.8%)
**RUNX1**	4 (14.8%)
**NPM1**	4 (14.8%)
**SRSF2**	3 (11.1%)
**SF3B1**	3 (11.1%)
**FLT3**	3 (11.1%)
**IDH1/2**	4 (14.8%)

Abbreviations: IQR, interquartile range; ECOG, Eastern Cooperative Oncology Group; IPSS-R, Revised International Prognostic Scoring System; IPSS-M, Molecular International Prognostic Scoring System; RBC, red blood cell (transfusion dependence).

**Table 2:** Logistic regression analysis for factors associated with overall response (ORR) and complete remission (CR) rates.

	Overall Response			Complete Response		
		Multivariate			Multivariate	
**Characteristics**	Univariate *p*-value	OR (95%CI)	*p*-value	Univariate *p*-value	OR (95%CI)	*p*-value
**Gender**	0.525			0.639		
**Age**	0.445			0.953		
**LDH**	0.695			0.584		
**SF**	0.211			0.438		
**WBC**	0.191	1.568 (0.507–4.851)	0.435	0.867		
**N**	0.169	0.496 (0.094–2.621)	0.409	0.946		
**HB**	0.092	0.954 (0.877–1.037)	0.269	0.389		
**PLT**	0.900			0.126	1.006 (0.992–1.021)	0.411
**BM BLAST**	0.851			0.891		
**IPSS-R**	0.190	0.352 (0.022–5.597)	0.459	0.571		
**cytogenetic**	1.000			0.292		
**DMNT3A**	0.865			0.324		
**ASXL1**	0.742			0.856		
**TP53**	1.000			0.197	1.386 (0.073–26.22)	0.828
**TET2**	0.999			0.999		
**NPM1**	0.999			0.109	0.168 (0.012–2.326)	0.183
**IDH1/2**	0.999			0.109	0.260 (0.018–3.824)	0.326
**RUNX1**	0.999			0.633		
**FLT3**	0.999			0.265		
**SF3B1**	0.343			0.927		
**SRSF2**	0.999			0.927		

Abbreviations: OR (for response), overall response; CR, complete remission; OR, Odds Ratio, CI, confidence interval; IPSS-R, Revised International Prognostic Scoring System; LDH, lactate dehydrogenase; SF, serum ferritin; WBC, white blood cell; N, neutrophils; HB, hemoglobin; PLT, platelets; mutation statuses refer to gene mutations present vs. absent.

**Table 3:** Cox proportional hazards model for overall survival.

	Overall Survival		
	Univariate	Multivariate	
**Characteristics**	*p*-value	HR (95%CI)	*p*-value
**Gender**	0.957		
**Age**	0.053	1.022 (0.930–1.123)	0.652
**LDH**	0.637		
**SF**	0.625		
**WBC**	0.465		
**N**	0.110	1.413 (1.066–1.872)	0.016
**HB**	0.388		
**PLT**	0.159	0.997 (0.986–1.009)	0.660
**BM BLAST**	0.018	1.236 (0.277–5.521)	0.781
**IPSS-R**	0.205		
**Cytogenetic**	0.047	0.069 (0.005–0.871)	0.039
**DMNT3A**	0.422		
**ASXL1**	0.625		
**TP53**	<0.001	128.908 (4.832–3438.954)	0.004
**TET2**	0.478		
**NPM1**	0.318		
**IDH1/2**	0.350		
**HSCT**	0.059	0.158 (0.016–1.566)	0.115
**OR**	0.455		
**CR**	0.130	1.181 (0.170–8.201)	0.866

Abbreviations: HR, hazard ratio; CI, confidence interval; IPSS-R, Revised International Prognostic Scoring System; WBC, white blood cell; N, neutrophils; PLT, platelets; HSCT, hematopoietic stem cell transplantation; CR, complete remission.

**Table 4:** Treatment-emergent adverse events (TEAEs) in 28 patients.

TEAE	Grade1/2 N (%)	Grade3/4 N (%)	Any Grade N (%)
**Hematological**			
**Leukopenia**	1 (3.6%)	26 (92.9%)	27 (96.4%)
**Neutropenia**	1 (3.6%)	27 (96.4%)	28 (100.0%)
**Anemia**	4 (14.3%)	20 (71.4%)	24 (85.7%)
**Thrombocytopenia**	9 (32.1%)	18 (64.3%)	27 (96.4%)
**Febrile neutropenia**	0 (0.0%)	14 (50.0%)	14 (50.0%)
**Non-hematological**			
**Pneumonia**	0 (0.0%)	10 (35.7%)	10 (35.7%)
**Nausea**	13 (46.4%)	0 (0.0%)	13 (46.4%)
**Vomiting**	3 (10.7%)	0 (0.0%)	3 (10.7%)
**Constipation**	9 (32.1%)	0 (0.0%)	9 (32.1%)
**Diarrhea**	2 (7.1%)	0 (0.0%)	2 (7.1%)
**Liver injury**	3 (10.7%)	0 (0.0%)	3 (10.7%)
**Renal injury**	3 (10.7%)	0 (0.0%)	3 (10.7%)
**Cardiotoxicity**	3 (10.7%)	0 (0.0%)	3 (10.7%)
**Skin/soft tissue infection**	1 (3.6%)	0 (0.0%)	1 (3.6%)
**Sepsis**	0 (0.0%)	1 (3.6%)	1 (3.6%)
**Tumor lysis syndrome**	0 (0.0%)	0 (0.0%)	0 (0.0%)

Abbreviations: TEAE, treatment-emergent adverse event; n, number of patients. Grading per NCI CTCAE v5.0.

## Data Availability

The datasets used and/or analysed during the current study are available from the corresponding author on reasonable request.

## Abbreviations

AML	Acute myeloid leukemia
AZA	Azacitidine (5-azacitidine)
BCL-2	B-cell lymphoma 2 (anti-apoptotic protein)
CR	Complete remission
HR-MDS	Higher-risk myelodysplastic syndromes
HSCT	Hematopoietic stem cell transplantation
ITD	Internal tandem duplication
IWG	International Working Group
MDS	Myelodysplastic syndromes
NCI CTCAE	National Cancer Institute Common Terminology Criteria for Adverse Events
NGS	Next-generation sequencing
OR	Overall response; also used as abbreviation for odds ratio (statistical term)
ORR	Objective response rate (overall response rate)
OS	Overall survival
PR	Partial remission
VA	Venetoclax plus azacitidine (combination regimen)
VAF	Variant allele frequency
